# Endoscopic Management of Gastric Volvulus in an Elderly Patient With Multiple Comorbidities: A Case Report

**DOI:** 10.7759/cureus.63799

**Published:** 2024-07-04

**Authors:** Laura Akiki, Abed AlRaouf Kawtharani, Antoine Abou Rached, Antoine Semaan, Antoine S Geagea, Antoine Abi Abboud

**Affiliations:** 1 Gastroenterology and Hepatology, Faculty of Medicine, Lebanese University, Beirut, LBN; 2 Internal Medicine - Gastroenetrology, Faculty of Medicine, Lebanese University, Beirut, LBN; 3 Anesthesia, Faculty of Medicine, Lebanese University, Beirut, LBN; 4 Gastroenterology and Hepatology, Lebanese Hospital Geitaoui University Medical Center, Beirut, LBN; 5 Gastroenterology and Hepatology, Lebanese University, Beirut, LBN

**Keywords:** derotation, gastroscopy, borchardt’s triad, elderly, gastric volvulus

## Abstract

Gastric volvulus is a rare condition whose incidence remains largely unknown. Unless actively considered by healthcare providers, the diagnosis of gastric volvulus, which can lead to significant morbidity and mortality, may be overlooked. This condition can manifest in either acute or chronic forms, presenting with diverse symptoms. Notably, the presence of a hiatal hernia alongside persistent vomiting despite initial antiemetic therapy should raise suspicion for gastric volvulus, even if the patient appears clinically stable. Acute gastric volvulus is usually managed surgically. Here, we describe the case of an elderly male who was diagnosed with acute gastric volvulus and was treated conservatively with endoscopy.

## Introduction

Gastric volvulus is defined by the abnormal rotation of the stomach along its longitudinal or transverse axis [1]. This condition can be primary in 10% to 30% of cases, often attributable to abnormalities in gastric ligaments, or as a secondary complication of splenic, gastric, or diaphragmatic disorder with the paraesophageal hernia being the most common cause in both children and adults [2]. Despite its significance, gastric volvulus is usually underdiagnosed with an estimated incidence of approximately 1/10,000 hospital admissions [3]. Notably, it exhibits no sex or race predilection and commonly affects adults over 50 years of age [1,2].

The acute presentation of gastric volvulus is a very rare clinical entity occurring in merely 4% of patients with hiatal hernia [4] and carries a high risk of strangulation, potentially leading to necrosis and perforation with a mortality rate reaching 30%-50%. Consequently, prompt diagnosis and early management are crucial [5].

Although surgery represents the gold standard treatment of gastric volvulus, conservative management is to be considered in selected cases [6].

We hereby present the case of an elderly comorbid patient who presented with acute gastric volvulus that was successfully managed conservatively with gastroscopy.

## Case presentation

An 85-year-old man presented to the ED with severe epigastric pain of three days duration. The pain was sharp, constant, non-radiating, and was barely relieved by over-the-counter (OTC) painkillers. It was associated with postprandial nausea and vomiting that led to a decrease in his per os (PO) intake. He denied having fever, chills, or any change in bowel habits.

The patient’s history is relevant for hypertension, diabetes mellitus, dyslipidemia, coronary artery disease, and ischemic cerebral vascular accident with a residual right hemiplegia. His home medications include metformin, statin, and dual anti-platelets.

In the ED, his vital signs were normal, with mild tachycardia noted. On physical examination, the patient appeared anxious and restless, lying in severe pain, with dry skin and oral mucosa. No icterus was observed. An abdominal examination revealed a soft abdomen with tenderness upon superficial and deep palpation of the epigastric region and a negative Murphy sign.

Laboratory tests revealed mild leukocytosis (WBC of 11.0 x 109 /L with 74% neutrophils), an isolated elevation in alkaline phosphatase to 147 international units per liter (IU/L) (upper limit is 90 IU/L), and a mildly elevated lipase of 233 U/L (upper limit is 160 U/L). The rest of her lab results (hemoglobin, platelets, creatinine, electrolytes, CRP, and cardiac enzymes) were within normal range.

**Figure 1:**
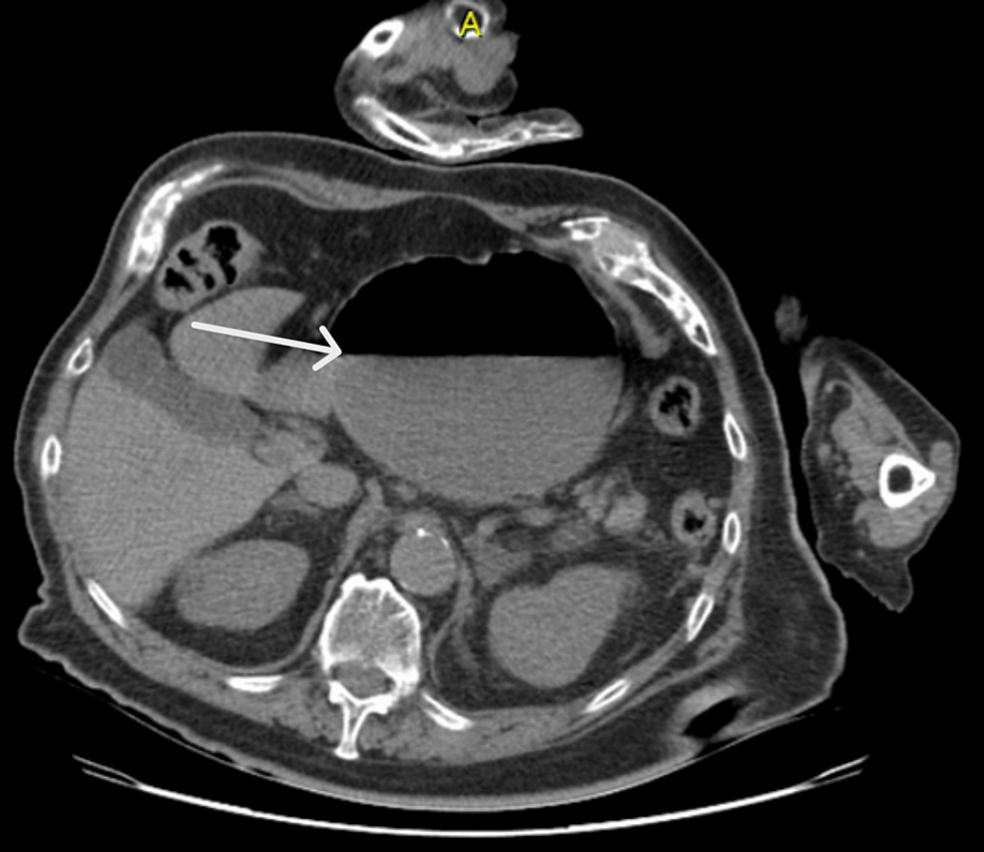
CT scan of the abdomen in the axial plane, showing a distended stomach with a double-pouch appearance and air-fluid levels (white arrow)

**Figure 2:**
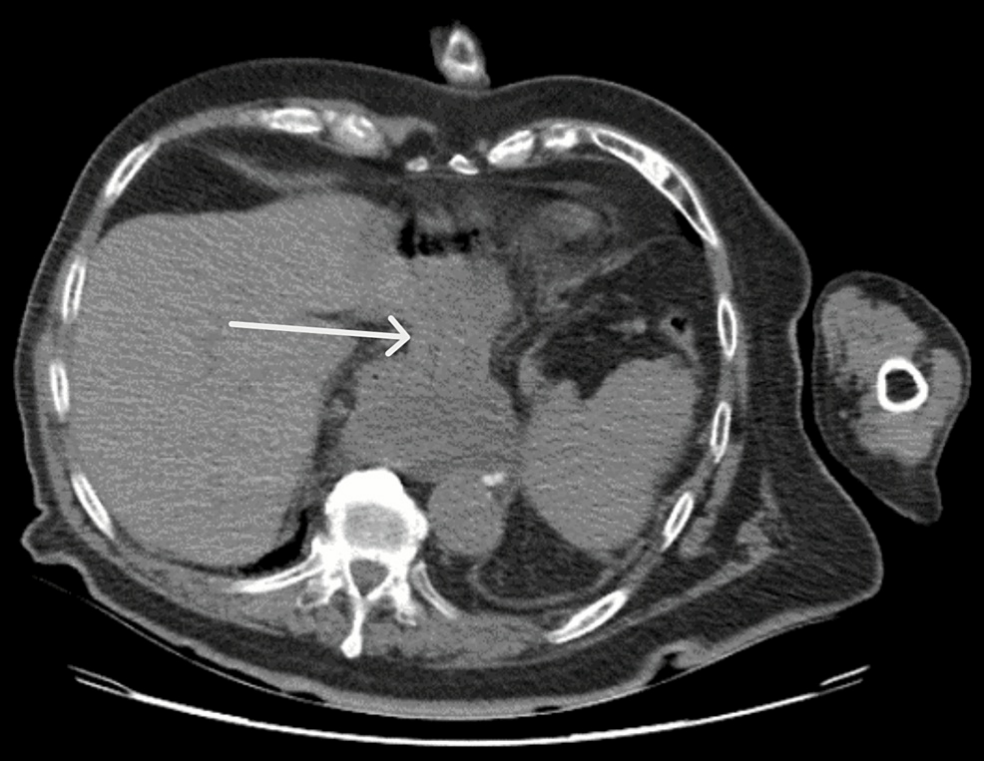
CT scan of the abdomen in the axial plane, showing twisting of the gastric pouch at the level of the diaphragmatic opening

The patient was started on IV hydration, symptomatic treatment with painkillers and antiemetics, and was kept nil per os (NPO). A contrast-enhanced CT of the abdomen showed dilatation of the gastric pouch with an air-fluid level and a double-pouch appearance, along with twisting of the gastric pouch at the diaphragmatic opening, suggesting gastric volvulus. No other significant findings were observed (Figures 1-2).

The diagnosis of acute gastric volvulus was made, and the case was initially discussed with the surgical team. However, given the patient’s advanced age and multiple comorbidities, a multidisciplinary decision was made for medical management.

**Figure 3:**
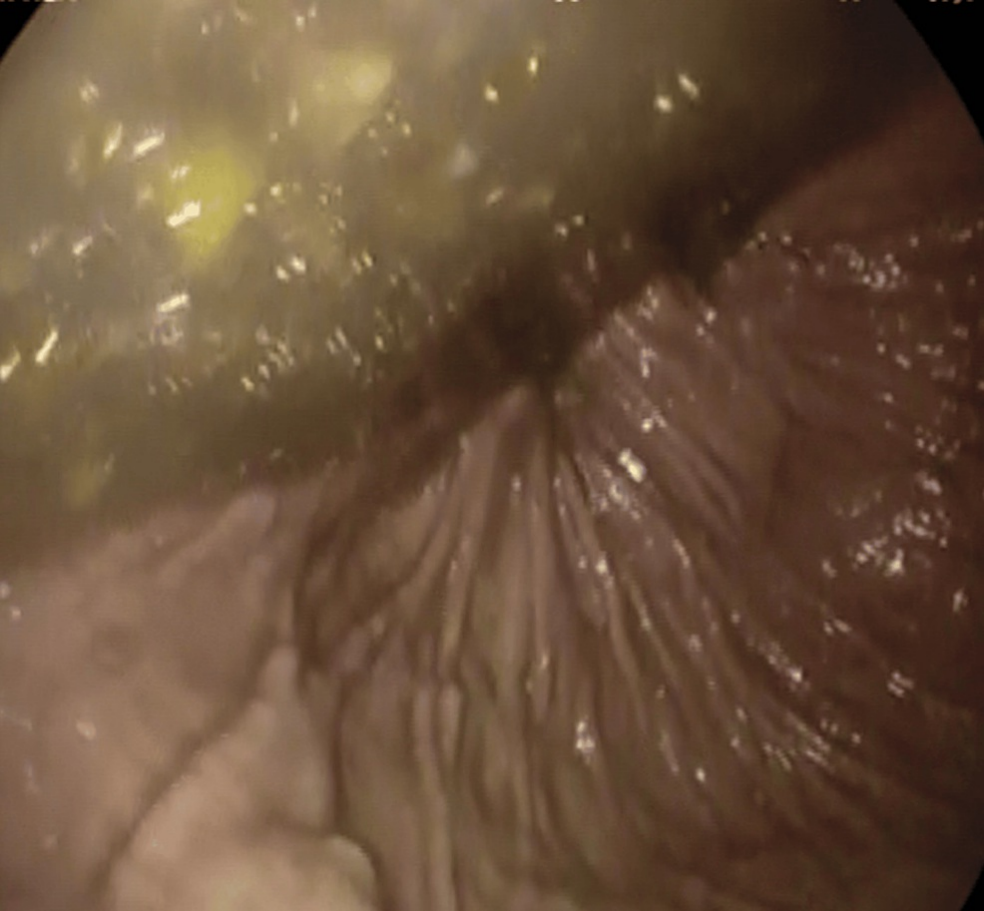
Gastroscopy showing residual food in the stomach

**Figure 4:**
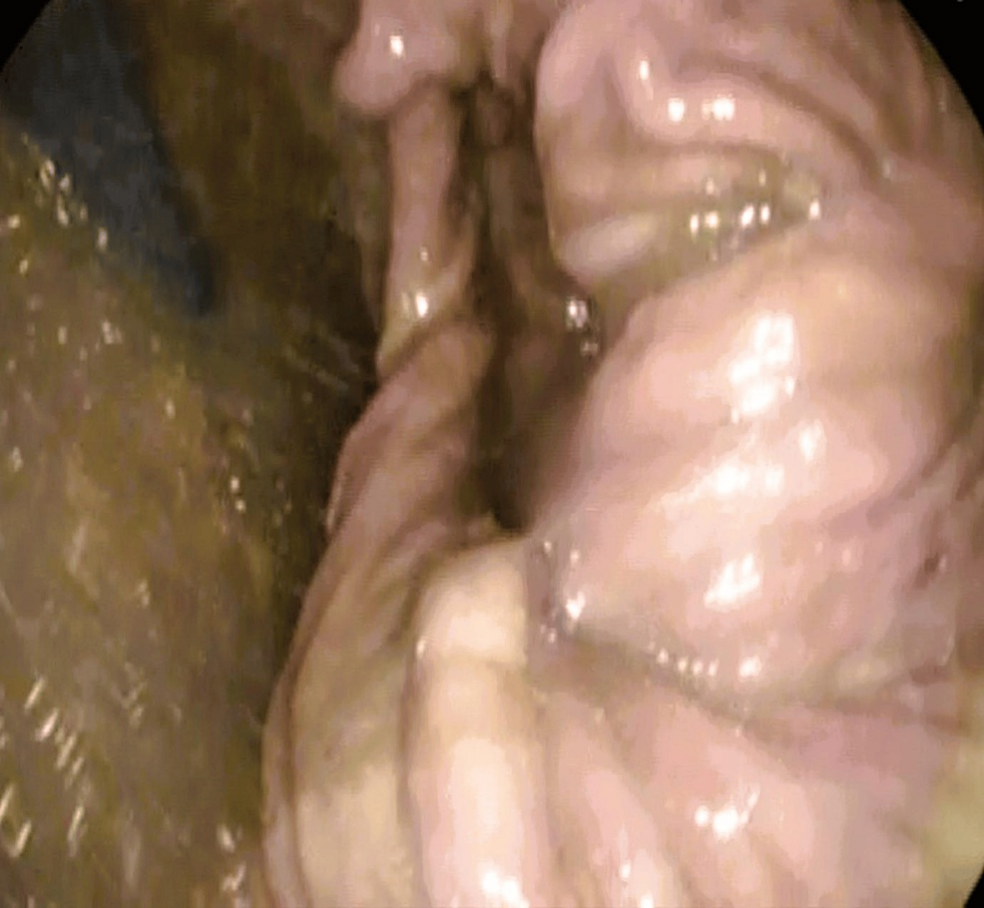
Gastric volvulus with paraesophageal hernia seen

A gastroscopy was done and showed food residue with no evidence of gastric necrosis (Figure 3). The gastric volvulus with paraesophageal hernia was seen (Figure 4), and a derotation was done by advancing the scope in an anticlockwise manner, and suction of the residual fluid was done.

**Figure 5:**
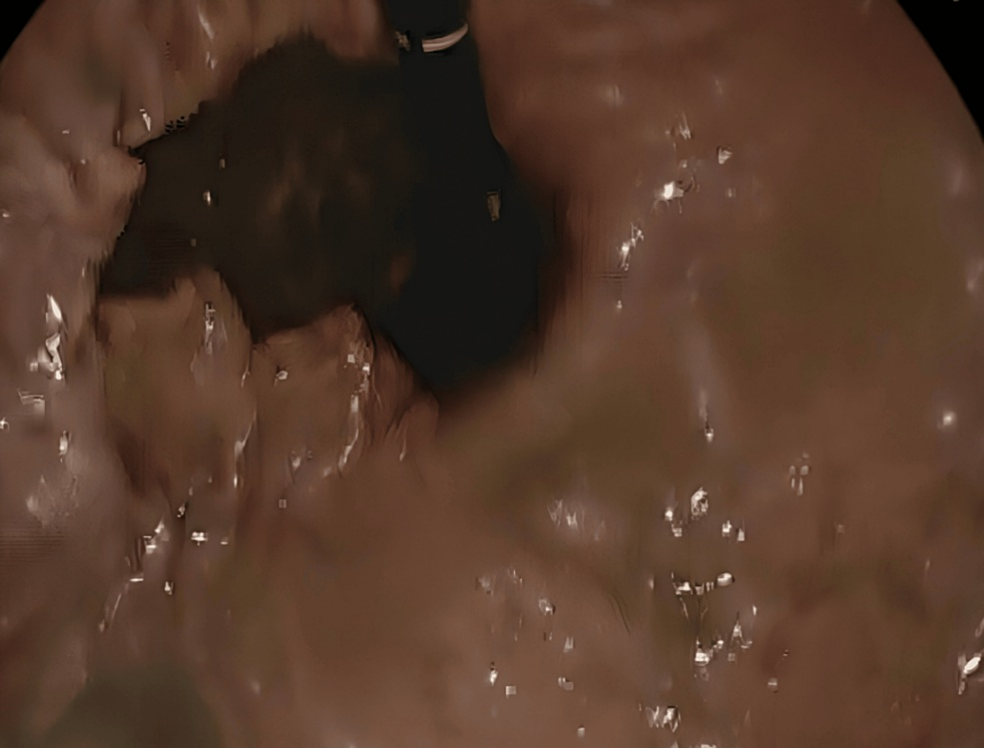
Retroflex view showing the stomach after the derotation of the gastric volvulus

The gastric volvulus was successfully reduced (Figure 5).

Clinical improvement was observed following gastroscopy. The patient was admitted to the regular floor with the oral diet resumed the next day. The risk of recurrence was explained to the patient and his family, who declined any surgical procedure.

## Discussion

Gastric volvulus is characterized by a variable clinical symptom ranging from an acute abdomen to chronic nonspecific gastrointestinal symptoms. Borchardt’s triad, which is seen in the acute presentation, is defined by severe epigastric pain, non-productive vomiting, and inability to pass a nasogastric (NG) tube. This triad is found in about 75% of cases [7].

On the other hand, recurrent nonspecific symptoms, such as retching, acid reflux, postprandial fullness, and chronic abdominal pain, are the main symptoms in patients with chronic gastric volvulus [8]. The incidence is not well reported in the literature because of the association of gastric volvulus with hiatal hernia [9].

Gastric volvulus can be classified into two subtypes based on the axis of rotation: organoaxial and mesenteroaxial. The former is the most common type, wherein the stomach rotates around the patient's vertical axis. It is mainly caused by diaphragmatic hernia, pyloric obstruction, adhesions, or enlarged esophageal hernia. The latter accounts for about one-third of all torsions, and it is idiopathic in 37% of cases. Moreover, in this case, the stomach rotates around the horizontal axis [10].

Because of lexical inconsistencies and overlapping entities, the pathophysiology of gastric volvulus has not been well understood [9].

The role of paraesophageal hernia in the pathophysiology of gastric volvulus, the concept of an upside-down stomach, and the imaging findings are subjected to several misconceptions [11]. Multiple complications can be observed in patients with acute gastric volvulus ranging from dehydration, aspiration, and pneumonia, to more severe ones with ischemia, perforation, pancreatitis, pancreatic necrosis, and splenic rupture [5].

Diagnosing gastric volvulus is challenging, primarily because of its rarity and variable presentation. Therefore, maintaining a high index of suspicion is paramount for timely identification [12,13]. A meticulous history-taking process is essential to recognize patients with chronic large hiatal hernias and other diaphragmatic abnormalities predisposing them to this condition. Additionally, clinical and radiological findings play crucial roles in establishing the diagnosis [14].

While a barium swallow remains the gold standard for diagnosing gastric volvulus because of its high sensitivity and specificity, its feasibility in the ED setting is limited [15]. Nonetheless, when available, it serves as a valuable diagnostic tool, aiding in confirming the diagnosis and guiding further management.

An abdominal CT scan is a valuable tool as it provides a definitive diagnosis, assesses complications, and helps in guiding the surgical intervention. However, the radiological findings on the abdominal CT are variable, making the gastric volvulus a challenging condition to diagnose with a CT scan [16].

In a single-center retrospective study conducted by Mazaheri et al. on 30 patients with gastric volvulus proven surgically, in whom a CT scan was done preoperatively, the authors showed substantial interobserver agreement (90%) and fair accuracy in the diagnosis of gastric volvulus among radiologists [17].

Variable CT features are reported such as severe gastric distention, gastric antrum in the left hemithorax, antro-pyloric junction above the gastroesophageal junction, greater gastric curvature superior and to the right of the lesser curvature, transition point at the pylorus and stenosis at the hernia neck. Moreover, a CT scan can detect complications related to the gastric volvulus like the presence of peri gastric fluid, gastric wall hypoenhancement, pneumatosis, gastric wall edema, pneumoperitoneum, celiac occlusion, and pleural effusion, all of which can be seen in case of gastric ischemia [16].

Concerning the management of gastric volvulus, Zuiki et al., recommend early endoscopy to search for gastric wall ischemia [17].

In the case of acute gastric volvulus, emergent surgical treatment is required in surgically fit patients either by laparoscopy or laparotomy for the reduction and decompression of the stomach, followed by gastropexy to prevent recurrence. In case of gastric necrosis, gastrectomy should be done [18]. Urgent abdominal exploration is mandatory in cases when gastric decompression fails by NG tube insertion or endoscopically [6].

A surgical repair of the anatomic defect is also needed in case of secondary gastric volvulus, and the exception to this rule are patients with multiple medical comorbidities in whom gastric derotation and fixation are done successfully by endoscopy [6].

In patients with chronic volvulus, the laparoscopic approach is the gold standard treatment; however, conservative management is considered the safest option [6].

In elderly patients with acute gastric volvulus who are unfit for surgery, an endoscopic detorsion is a viable option as demonstrated in our case. In patients with poor oral intake, percutaneous endoscopic gastrostomy (PEG) insertion has been an option for both gastric fixation and enteral nutrition [17].

Concerning complications related to endoscopic therapy, there is a significant risk of gastric perforation in addition to a risk of recurrence because of inadequate fixation and the persistence of the underlying predisposing factors [19].

In some elderly patients, endoscopic or laparoscopic gastropexy may be enough. However, further larger studies are needed to determine the definitive management technique in this category of patients [17].

## Conclusions

Gastric volvulus is a rare entity that can manifest and present with variable symptoms, posing challenges in diagnosis. A thorough history-taking in conjunction with imaging can help in the effective diagnosis and guide the appropriate treatment, with surgical therapy being the best approachpatientsient with acute volvulus. This case report highlights the role of conservative management in patients presenting with acute gastric volvulus who have multiple medical comorbidities are and considered unfit for surgical intervention. Nevertheless, large studies are needed to establish a definitive management for this category of patients.
